# Association between self-reported difficulty in chewing or swallowing and frailty in older adults: A retrospective cohort study

**DOI:** 10.1007/s11357-024-01325-7

**Published:** 2024-08-30

**Authors:** So Sato, Yusuke Sasabuchi, Akira Okada, Hideo Yasunaga

**Affiliations:** 1 Department of Clinical Epidemiology and Health Economics, Graduate School of Medicine, The University of Tokyo, 7-3-1, Hongo, Bunkyo-Ku, Tokyo, 1130033 Japan; 2 Department of Real-World Evidence, Graduate School of Medicine, The University of Tokyo, Tokyo, Japan; 3 Department of Prevention of Diabetes and Lifestyle-Related Diseases, Graduate School of Medicine, The University of Tokyo, Tokyo, Japan

**Keywords:** Self-reported oral function, Aspiration pneumonia, Frailty, Chewing, Swallowing

## Abstract

Oral frailty can contribute to physical and mental health disorders. Previous research has shown an association between frailty and self-reported difficulty in chewing or swallowing. However, their combined assessment has obscured their specific impact on frailty-related outcomes. To investigate the independent associations between difficulty in chewing or swallowing and 1-year frailty outcomes, while also examining their interactions. This retrospective cohort study utilized the DeSC database to identify older adults aged ≥ 75 years who underwent health checkups between April 2014 and November 2022. Multivariate Cox regression analyses were conducted to assess the association between self-reported difficulty in chewing or swallowing and outcomes, including hospitalization due to aspiration pneumonia, all-cause hospitalization, and all-cause mortality within 1 year. Interactions between chewing and swallowing difficulties were also evaluated. Among 359,111 older adults, 39.0% reported oral function difficulties. Swallowing difficulty alone lacked significant outcome association. However, chewing difficulty alone was significantly associated with higher risks of hospitalization due to aspiration pneumonia (hazard ratio (HR), 1.35; 95% confidence interval (CI), 1.15–1.58; P < 0.001), all-cause hospitalization (HR, 1.08; 95% CI, 1.05–1.11; P < 0.001), and all-cause mortality (HR, 1.28; 95% CI, 1.14–1.44; P < 0.001) compared with no self-reported difficulty. A significant positive interaction between self-reported difficulty in chewing and swallowing was observed for all-cause mortality (P = 0.009). Self-reported difficulty in chewing was significantly associated with higher risks of hospitalization due to aspiration pneumonia, all-cause hospitalization, and all-cause mortality among older adults. Chewing and swallowing difficulties showed a synergistic effect, significantly increasing all-cause mortality risk.

**Supplementary Information:**

The online version contains supplementary material available at 10.1007/s11357-024-01325-7.

## Introduction

Oral frailty encompasses various phenomena and processes marked by vulnerability in oral health due to age-related changes in different oral health conditions, including the number of teeth, oral hygiene, and oral functions [1]. This vulnerability includes diminished interest in oral health and decreased physical and mental reserve capacity, leading to a decline in eating function and eventually contributing to physical and mental disorders [1, 2].

Chewing and swallowing are independent components of oral function: chewing prepares food for swallowing, while swallowing transports food for digestion [3]. Numerous studies have shown that self-reported difficulties in chewing and swallowing are associated with the progression of frailty [4–18], including the development of dysphagia [17, 18], and increased mortality [4]. However, most of these studies considered chewing and swallowing as a single function. No study has analyzed both self-reported chewing and swallowing functions in the same individuals as independent predictors of frailty. Therefore, it remains unclear which of chewing or swallowing is more relevant to frailty. Japan has introduced a unique 15-item brief questionnaire to screen for frailty among older adults aged ≥ 75 years, which includes two items on self-reported difficulties in chewing and swallowing [19]. However, the questionnaire’s ability to screen for oral frailty remains unknown.

This study utilized a large database of health checkups and administrative claims data, including the unique Japanese 15-item brief questionnaire for screening frailty, which specifically includes two items on self-reported difficulty in chewing and swallowing. The study aimed to investigate the independent associations between difficulty in chewing or swallowing and 1-year outcomes related to frailty using these two items. The study also investigated the interaction between self-reported difficulty in chewing and swallowing.

## Methods

### Data source

This study utilized the commercially available DeSC database (DeSC Healthcare, Inc., Tokyo, Japan), which contains administrative claims and health checkup data. Detailed information regarding this database is available elsewhere [20]. In summary, the database comprises health insurance claims from three types of insurers: (i) National Health Insurance for non-employees and individual proprietors, (ii) health insurance for large corporate employees, and (iii) Advanced Elderly Medical Service System for those aged ≥ 75 years. Thus, the DeSC database encompasses individuals across various age groups, including young, middle-aged, and older adults. The DeSC database includes data for approximately 12,500,000 individuals, closely mirroring the Japanese population estimates in age distribution [20].

Medical claims data comprising both outpatient and inpatient records were anonymized and maintained at the individual level. This encompasses unique identifiers; birth month; sex; diagnoses coded according to the International Classification of Diseases, 10th Revision (ICD-10) codes; procedures based on the original Japanese identification system; pharmaceutical dispensations recorded using the Anatomical Therapeutic Chemical (ATC) Classification System; and dates of insurance enrollment and disenrollment.

Health check-up data were also included in the database. During their annual health checkups, older adults aged ≥ 75 years complete a Japanese unique 15-item brief questionnaire for screening frailty, known as Late-Stage Elderly Questionnaire [19]. This self-administered questionnaire organized into ten categories: health status, mental health status, dietary habits, oral function, weight change, physical function and falls, cognitive function, smoking, social engagement, and social support. The oral function section of the questionnaire includes two questions on self-reported difficulties in chewing ("Do you have any difficulties eating tough foods compared to 6 months ago?") and swallowing ("Have you choked on your tea or soup recently?").

### Study design and participant selection

This retrospective cohort study utilized data collected between April 2014 and November 2022. The index date for each participant was defined as the day of their initial health checkup during the observation period. The inclusion criteria comprised individuals who (i) underwent health checkups for adults aged ≥ 75 years, (ii) provided complete responses to the oral function section of the Late-Stage Elderly Questionnaire, and (iii) were enrolled in the DeSC database within 1 year before the health checkup, enabling a retrospective 1-year observation period before the index date as a lookback period. Individuals who (i) were diagnosed with aspiration pneumonia (ICD-10 code: J69x), (ii) had undergone gastrostomy, or (iii) had incomplete body mass index data during their lookback period were excluded. Eligible individuals were followed from the index date until hospitalization due to aspiration pneumonia, disenrollment in the DeSC database, death, end of the study period, or 1 year after the index date, whichever came first.

### Exposure of interest

Self-reported difficulty in chewing was identified when older adults responded “yes” to the question, "Do you have any difficulties eating tough foods compared to 6 months ago?" Similarly, self-reported difficulty in swallowing was identified when older adults responded “yes” to the question, "Have you choked on your tea or soup recently?".

### Outcomes and covariates

The primary outcome in this study was hospitalization due to aspiration pneumonia within 1 year of the health checkup, as defined by ICD-10 codes (J69x). Secondary outcomes included all-cause hospitalization and mortality within 1 year of the health checkup.

Covariates included age (75–79, 80–84, 85–89, 90–94, and ≥ 95 years); sex; body mass index (< 18.5, 18.5–25.0, 25.0–30.0, and ≥ 30.0 kg/m^2^); smoking status (non-smoker, current smoker, and past smoker); Charlson comorbidity index (0–2, 3–4, and ≥ 5) [21], which assessed the severity of comorbidities; the number of prescribed medications, reflecting polypharmacy (0–4: non-polypharmacy, 5–9: polypharmacy, and ≥ 10: hyper-polypharmacy) [22]; comorbidities, medications; and cerebrovascular rehabilitation and nasal feeding, which are medical procedures associated with dysphagia or aspiration pneumonia [23–25]. These variables were selected based on prior research on dysphagia or aspiration pneumonia [17, 18, 23–25]. Online Resource 1 provides further details. Following a previous study [22], data on 88 frequently prescribed medications and those associated with potential adverse effects in older adults were extracted. Short-acting non-benzodiazepine hypnotics and nitric acid medicines were excluded because they are not covered by the Japanese public health insurance, resulting in 86 medication categories [26]. Polypharmacy was determined by calculating the number of prescribed drug classes from the 86 available categories for each individual. Comorbidities related to dysphagia or aspiration pneumonia were identified if they occurred within 365 days before the index date, whereas the number of prescribed medications was determined within 30 days before the index date [27].

### Statistical analysis

Baseline characteristics are summarized using proportions for binary variables and means with standard deviations for continuous variables. These characteristics were compared between individuals with and without self-reported difficulties in oral function.

Multivariable Cox regression analyses were conducted to evaluate the association between self-reported difficulties in chewing and swallowing and each outcome within 1 year. Self-reported difficulties in chewing and swallowing were treated as independent factors. The dependent variable in the Cox regression models was time from the day of the health checkup to the day of the occurrence of each outcome (specifically, hospitalization due to aspiration as the primary outcome and all-cause hospitalization and death as the secondary outcomes). These analyses also assessed the interaction between self-reported difficulties in chewing and swallowing. All the aforementioned covariates were included for adjustment in the multivariate Cox regression analyses. Hazard ratios (HRs) were calculated to estimate the association between self-reported difficulty in chewing or swallowing and each outcome, with consideration given to the interactions. The reference group comprised individuals without self-reported difficulties in oral function.

In the subgroup analysis, the association between self-reported difficulty in chewing and outcomes was assessed in subgroups with and without self-reported difficulty in swallowing, considering interactions.

Statistical significance was set at P < 0.05. All statistical analyses were performed using Stata SE software, version 18.0 (StataCorp, College Station, TX, USA).

## Results

**Fig. 1:**
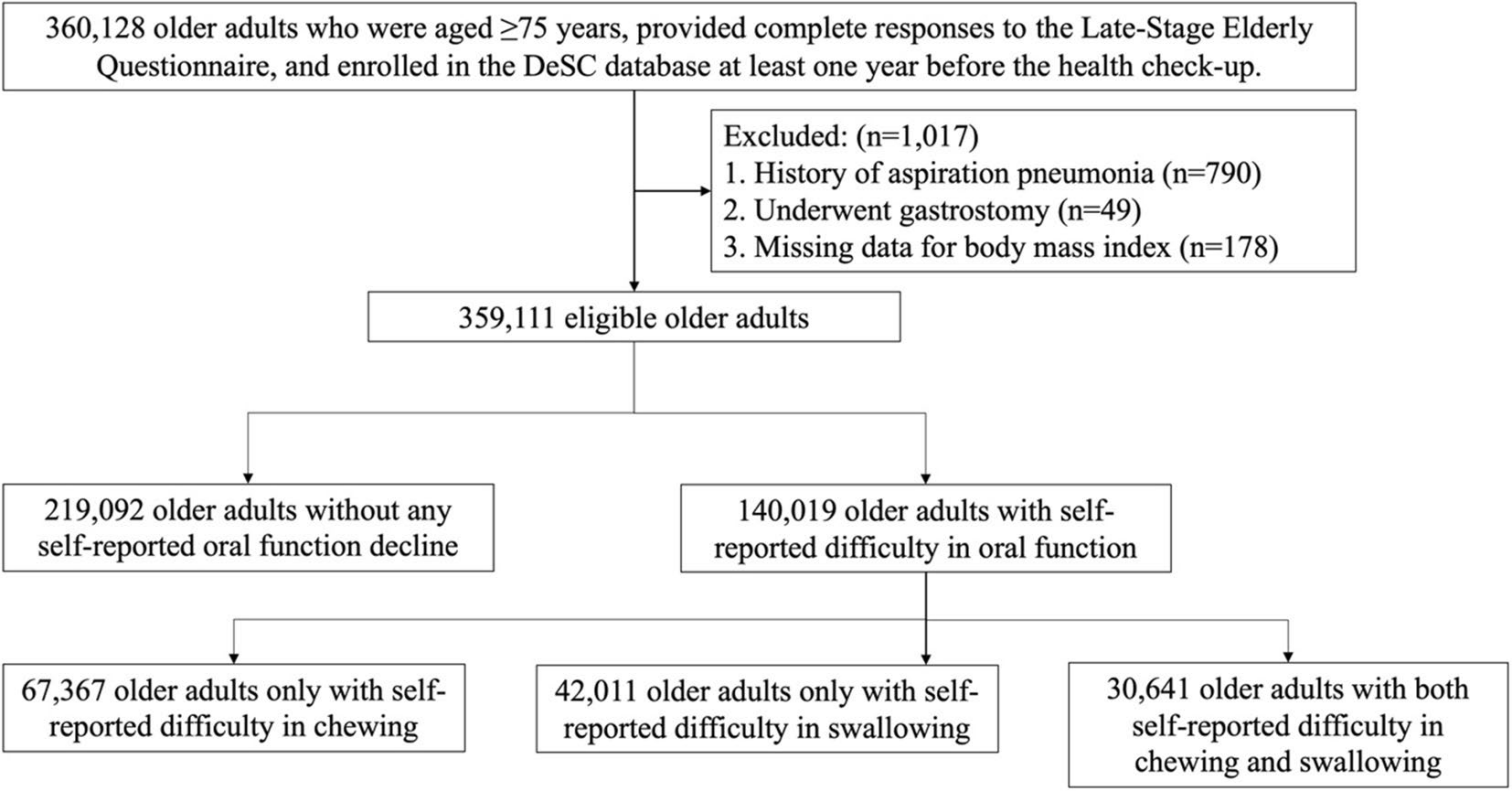
Flowchart of the selection of study participants

After applying the inclusion and exclusion criteria, 359,111 older adults were identified between April 2014 and November 2022 (Fig. 1). Aspiration pneumonia resulted in the hospitalization of 994 (0.3%) older adults, while all-cause hospitalization occurred in 37,218 (10.4%) older adults, and 1,852 (0.5%) died. The median follow-up duration was 245 days (interquartile range, 132–363 days; mean, 256 days; standard deviation, 167 days). Out of 134,429 person-years of follow-up, the incidence rate of hospitalization due to aspiration pneumonia was 7.4 per 1,000 person-years (95% confidence interval [CI], 6.9 to 7.9).

Table 1 displays the baseline characteristics of older adults with and without self-reported difficulties in oral function. Among the participants, 39.0% reported difficulties in oral function. This group consisted of older adults who reported difficulties only in chewing (48.1%), only in swallowing (30.0%), and in both chewing and swallowing (21.9%). Older adults who reported difficulties in oral function tended to be older, female, have lower body mass indexes, have higher Charlson Comorbidity Index scores, and be more likely to have current or past smoking habits than those without any self-reported difficulty in oral function. Additionally, they were more likely to be subject to polypharmacy, have comorbidities (excluding diabetes), use medications, utilize cerebrovascular rehabilitation services, and have undergone nasal feeding compared with those without any self-reported difficulty in oral function.

**Table 1:** Characteristics of participating older adults

	Without difficulty in self-reported oral function N = 219,092		With difficulty in self-reported oral function N = 140,019		P-value
	n	(%)	n	(%)	
Age, years					< 0.001
75–79	116,220	(53.0%)	63,136	(45.1%)	
80–84	68,964	(31.5%)	45,706	(32.6%)	
85–89	26,629	(12.2%)	22,675	(16.2%)	
90–94	6,374	(2.9%)	7,068	(5.0%)	
≥ 95	905	(0.4%)	1,434	(1.0%)	
Male	97,240	(44.4%)	55,079	(39.3%)	< 0.001
Body mass index, kg/m^2^					< 0.001
< 18.5	14,774	(6.7%)	11,989	(8.6%)	
18.5–24.9	149,500	(68.2%)	94,462	(67.5%)	
25.0–29.9	49,549	(22.6%)	29,943	(21.4%)	
≥ 30.0	5,269	(2.4%)	3,625	(2.6%)	
Charlson comorbidity index					< 0.001
0–2	176,634	(80.6%)	107,571	(76.8%)	
3–4	34,840	(15.9%)	25,907	(18.5%)	
≥ 5	7,618	(3.5%)	6,541	(4.7%)	
Smoking habit					< 0.001
Non-smoker	170,118	(77.7%)	107,686	(77.0%)	
Current smoker	10,828	(4.9%)	7,325	(5.2%)	
Past smoker	38,001	(17.4%)	24,921	(17.8%)	
Polypharmacy					< 0.001
No polypharmacy (0–4)	174,234	(79.5%)	104,862	(74.9%)	
Polypharmacy (5–9)	42,902	(19.6%)	33,186	(23.7%)	
Hyper-polypharmacy (≥ 10)	1,956	(0.9%)	1,971	(1.4%)	
Comorbid medical conditions					
Diabetes	65,927	(30.1%)	42,375	(30.3%)	0.270
Dementia	8,903	(4.1%)	7,075	(5.1%)	< 0.001
Parkinson's disease or Parkinsonian disorder	2,024	(0.9%)	2,290	(1.6%)	< 0.001
Hypertension	145,579	(66.4%)	94,943	(67.8%)	< 0.001
Angina pectoris	30,066	(13.7%)	22,122	(15.8%)	< 0.001
Atrial fibrillation	14,280	(6.5%)	9,914	(7.1%)	< 0.001
Heart failure	38,208	(17.4%)	28,493	(20.3%)	< 0.001
Cerebrovascular disease	46,227	(21.1%)	33,490	(23.9%)	< 0.001
Pneumonia	6,124	(2.8%)	4,779	(3.4%)	< 0.001
Asthma	23,799	(10.9%)	18,398	(13.1%)	< 0.001
Gastroesophageal reflux disease	77,720	(35.5%)	58,050	(41.5%)	< 0.001
Renal failure	10,052	(4.6%)	7,059	(5.0%)	< 0.001
Eating and swallowing dysfunction	3,427	(1.6%)	2,819	(2.0%)	< 0.001
Drugs					
Antiepileptics	15,972	(7.3%)	12,396	(8.9%)	< 0.001
Anticholinergics	326	(0.1%)	347	(0.2%)	< 0.001
Hypnotics	54,546	(24.9%)	41,124	(29.4%)	< 0.001
Cerebrovascular rehabilitation	1,494	(0.7%)	1,275	(0.9%)	< 0.001
Nasal feeding	104	(0.0%)	67	(0.0%)	0.960
Self-reported difficulty in oral function					< 0.001
Without any self-reported difficulty in oral function	219,092	(100.0%)	0	(0.0%)	
Only self-reported difficulty in chewing	0	(0.0%)	67,367	(48.1%)	
Only self-reported difficulty in swallowing	0	(0.0%)	42,011	(30.0%)	
Both self-reported difficulty in chewing and swallowing	0	(0.0%)	30,641	(21.9%)	

Table 2 displays the results of the multivariate Cox regression analyses. Older adults with only self-reported difficulty in swallowing were not associated with a higher risk of hospitalization due to aspiration pneumonia (HR 1.10, 95% CI 0.89–1.36, P = 0.389), all-cause hospitalization (HR 1.01, 95% CI 0.97–1.04, P = 0.678), or all-cause mortality (HR 1.02, 95% CI 0.87–1.20, P = 0.776) compared with those without any self-reported difficulty in oral function. On the other hand, those with only self-reported difficulty in chewing were significantly associated with a higher risk of hospitalization due to aspiration pneumonia (HR 1.35, 95% CI 1.15–1.58, P < 0.001), all-cause hospitalization (HR 1.08, 95% CI 1.05–1.11, P < 0.001), and all-cause mortality (HR 1.28, 95% CI 1.14–1.44, P < 0.001), respectively, than those without any self-reported difficulty in oral function. The interactions were not significant for hospitalization due to aspiration pneumonia (P = 0.175) or all-cause hospitalization (P = 0.214). However, a significant positive interaction was observed for all-cause mortality (P = 0.009). The complete results for the multivariate Cox regression analyses are presented in Online Resources 2–4.

**Table 2:** Results of multivariate Cox regression analysis for frailty-related outcomes, considering interactions

Frailty-related outcomes according to each status of self-reported difficulty in oral function	HR	95% CI	P-value
Hospitalization due to aspiration pneumonia			
Only self-reported difficulty in chewing	1.35	1.15–1.58	< 0.001
Only self-reported difficulty in swallowing	1.10	0.89–1.36	0.390
Interaction between self-reported difficulty in chewing and swallowing	1.22	0.92–1.63	0.175
Both self-reported difficulty in chewing and swallowing	1.81	1.51–2.16	< 0.001
All-cause hospitalization			
Only self-reported difficulty in chewing	1.08	1.05–1.11	< 0.001
Only self-reported difficulty in swallowing	1.01	0.97–1.04	0.678
Interaction between self-reported difficulty in chewing and swallowing	1.03	0.98–1.09	0.214
Both self-reported difficulty in chewing and swallowing	1.12	1.09–1.16	< 0.001
All-cause mortality			
Only self-reported difficulty in chewing	1.28	1.14–1.44	< 0.001
Only self-reported difficulty in swallowing	1.02	0.87–1.20	0.777
Interaction between self-reported difficulty in chewing and swallowing	1.33	1.07–1.66	0.009
Both self-reported difficulty in chewing and swallowing	1.75	1.53–1.99	< 0.001

CI, confidence interval; HR, hazard ratio

Table 3 presents the HRs for the outcomes associated with self-reported difficulty in chewing in subgroups with and without self-reported difficulty in swallowing. In the subgroup with self-reported difficulty in swallowing, the HR for each outcome associated with self-reported difficulty in chewing was higher than that in the subgroup without self-reported difficulty in swallowing.

**Table 3:** Hazard ratios for frailty-related outcomes among older adults reporting difficulty in chewing, stratified by swallowing status

Frailty-related outcomes in older adults with self-reported difficulty in chewing	Without self-reported difficulty in swallowing			With self-reported difficulty in swallowing		
	HR	95% CI	P-value	HR	95% CI	P-value
Hospitalization due to aspiration pneumonia	1.35	1.15–1.58	< 0.001	1.65	1.29–2.10	< 0.001
All-cause hospitalization	1.08	1.05–1.11	< 0.001	1.12	1.07–1.17	< 0.001
All-cause mortality	1.28	1.14–1.44	< 0.001	1.71	1.42–2.05	< 0.001

CI, confidence interval; HR, hazard ratio

## Discussion

This retrospective cohort study demonstrated a significant association between self-reported difficulty in chewing and higher risks of hospitalization due to aspiration pneumonia, all-cause hospitalization, and all-cause mortality within 1 year. Additionally, a positive interaction was observed between self-reported difficulties in chewing and swallowing for all-cause mortality.

In this study, self-reported difficulties in oral function were significantly associated with a higher risk of frailty. This finding aligns with those of previous studies on self-reported oral problems [4–18]. Previous studies on oral frailty often employed objective assessment methods, such as examining occlusal force or counting the number of teeth, to evaluate oral function decline [28, 29]. However, self-reported oral function assessment may provide a simpler alternative, requiring fewer resources and costing less than objective methods. Therefore, self-reported oral function assessment may be more practical than objective methods for screening oral frailty, particularly when considering the incorporation of oral function evaluations into frailty assessment.

Our results showed a significant association between self-reported difficulty in chewing and frailty-related outcomes in older adults. However, the association between self-reported difficulty in swallowing and frailty-related outcomes was not significant. The mechanism underlying the association between chewing difficulty and a higher risk of frailty compared with swallowing is unclear. Two possible explanations could account for this association. First, individuals reporting difficulty in chewing may avoid consuming tough foods, such as meat, vegetables, and fruits. These foods are typically rich in essential nutrients, including proteins, vitamins, and fibers [30–32]. Consequently, such dietary restrictions may contribute to surrogate indicators of frailty, including malnutrition and unintended weight loss [10, 33]. Second, self-reported difficulty in chewing may reflect a broader decline in muscle strength [8, 34]. Thus, healthcare providers should prioritize older adults reporting difficulty in chewing over those reporting difficulty in swallowing when assessing frailty risk.

Our findings indicate a positive interaction between self-reported difficulties in chewing and swallowing for all-cause mortality. Self-reported difficulties in both chewing and swallowing mutually exacerbate all-cause mortality, emphasizing the importance of simultaneously assessing these factors. This finding aligns with the concept of oral frailty and highlights the need for comprehensive attention to oral health problems [1]. Specifically, our results suggest that the potential risk of mortality may be underestimated when evaluating older adults with self-reported swallowing difficulties. Therefore, concurrently evaluating difficulties in chewing and swallowing may enhance our understanding and management of frailty risk among older adults.

To the best of our knowledge, this is the first study to evaluate the independent associations of difficulty in chewing and swallowing with 1-year outcomes related to frailty. Furthermore, this study had the largest sample size among all studies that have examined the association between oral function and frailty [2].

This study had several limitations warranting consideration. First, because health checkups are not mandatory, participation bias may have favored healthier older adults. This selection bias may have affected the generalizability of our findings. Second, this study focused exclusively on Japanese residents, which may limit the broader applicability of the results to other nations. Third, our analysis was limited by the lack of detailed information in the DeSC database. The underlying reasons for self-reported difficulty in chewing or swallowing, such as tooth loss, were not captured. Future studies are warranted to explore the specific impact of factors underlying self-reported difficulty in chewing or swallowing on frailty outcomes. Fourth, the DeSC database lacks information on the results of clinical swallowing examinations. Therefore, there is a possibility of misclassification in identifying hospitalizations due to aspiration pneumonia, which could have biased the study's results.

In conclusion, this study highlights a significant association between self-reported difficulty in chewing and an elevated risk of hospitalization due to aspiration pneumonia, all-cause hospitalization, and all-cause mortality. Moreover, self-reported difficulties in chewing and swallowing mutually exacerbated all-cause mortality. Concurrently evaluating difficulties in chewing and swallowing may enhance our understanding and management of frailty risk among older adults.

## Supplementary Information

Below is the link to the electronic supplementary material.

Supplementary file1 (DOCX 33 KB)

## Data Availability

The datasets analyzed in this study are commercially available (DeSC Healthcare, Inc., providing the database).
