# Supplementary material for: Association between self-reported difficulty in chewing or swallowing and frailty in older adults: A retrospective cohort study
Source: GeroScience. 2024 Aug 30;47(3):3497–505. doi: 10.1007/s11357-024-01325-7 (PMC12181454; doi:10.1007/s11357-024-01325-7)
Supplement: Supplementary file 1 — Supplementary file1 (DOCX 33 KB) [file 11357_2024_1325_MOESM1_ESM.docx]

**Association Between Self-Reported Difficulty in Chewing or Swallowing and Frailty in Older Adults: A Retrospective Cohort Study**

**Online Resource**

**CONTENTS**

**Online Resource 1.** Details of study covariates.

**Online Resource 2.** Detailed results of multivariate Cox regression analysis for hospitalization due to aspiration pneumonia.

**Online Resource 3.** Detailed results of multivariate Cox regression analysis for all-cause hospitalization.

**Online Resource 4.** Detailed results of multivariate Cox regression analysis for all-cause mortality.

**Online Resource 1.** Details of study covariates.

| Patient characteristics | Comorbidities medical conditions related to dysphagia or aspiration pneumonia (ICD-10 codes) | Drugs related to dysphagia or aspiration pneumonia (ATC classification) | Medical procedure related to dysphagia or aspiration pneumonia (Japanese procedure code) |
| --- | --- | --- | --- |
| Age (75–79, 80–84, 85–89, 90–94, and ≥95 years)  Sex  Body mass index (<18.5, 18.5–25.0, 25.0–30.0, and ≥30.0 kg/m^2^)  Smoking habit (non-smoker, current smoker, and past smoker)  Charlson comorbidity index (0–2, 3–4, and ≥5)  Polypharmacy (0–4: non-polypharmacy, 5–9: polypharmacy, and ≥10: hyper-polypharmacy) | Diabetes (E10–E14)  Dementia (F00–F02)  Parkinson's disease or Parkinsonian disorder (G20–G22)  Hypertension (I10, I15)  Angina pectoris (I20)  Atrial fibrillation (I48)  Heart failure (I11, I50)  Cerebrovascular diseases (I60–I69)  Pneumonia (J12–J18, J84, J95.8)  Asthma (J45)  Gastroesophageal reflux disease (K21)  Renal failure (N17–N19)  Eating and swallowing dysfunction (R19.8) | Antiepileptics (N03)  Anticholinergics (N04A)  Hypnotics (N05C) | Rehabilitation for cerebrovascular disease (H001)  Nasal feeding (J120) |

ICD, International Classification of Diseases and Related Health Problems, 10th revision code; ATC, Anatomical Therapeutic Chemical.

**Online Resource 2.** Detailed results of multivariate Cox regression analysis for hospitalization due to aspiration pneumonia.

|  | Hazard ratio | 95% confidence interval | P-value |
| --- | --- | --- | --- |
| Self-reported difficulty in oral function |  |  |  |
| Without any self-reported difficulty in oral function | reference | | |
| Only self-reported difficulty in chewing | 1.35 | 1.15–1.58 | <0.001 |
| Only self-reported difficulty in swallowing | 1.10 | 0.89–1.36 | 0.389 |
| Both self-reported difficulty in chewing and swallowing | 1.81 | 1.52–2.16 | <0.001 |
| Age, years |  |  |  |
| 75–79 | reference | | |
| 80–84 | 2.40 | 1.99–2.89 | <0.001 |
| 85–89 | 4.55 | 3.74–5.52 | <0.001 |
| 90–94 | 9.03 | 7.23–11.29 | <0.001 |
| ≥95 | 18.79 | 13.92–25.37 | <0.001 |
| Male | 2.89 | 2.49–3.35 | <0.001 |
| Body mass index, kg/m^2^ |  |  |  |
| <18.5 | 2.47 | 2.09–2.93 | <0.001 |
| 18.5–24.9 | reference | | |
| 25.0-29.9 | 0.81 | 0.68–0.96 | 0.015 |
| ≥30.0 | 1.02 | 0.66–1.57 | 0.942 |
| Charlson comorbidity index |  |  |  |
| 0–2 | reference | | |
| 3–4 | 1.06 | 0.89–1.26 | 0.533 |
| ≥5 | 0.87 | 0.66–1.14 | 0.312 |
| Smoking habit |  |  |  |
| Non-smoker | reference | | |
| Current smoker | 1.13 | 0.86–1.48 | 0.377 |
| Past smoker | 1.14 | 0.97–1.33 | 0.108 |
| Polypharmacy |  |  |  |
| No polypharmacy (0–4) | reference | | |
| Polypharmacy (5–9) | 1.25 | 1.07–1.45 | 0.004 |
| Hyper-polypharmacy (≥10) | 1.75 | 1.20–2.57 | 0.004 |
| Comorbid medical conditions |  |  |  |
| Diabetes | 1.02 | 0.89–1.17 | 0.749 |
| Dementia | 2.56 | 2.16–3.04 | <0.001 |
| Parkinson's disease or Parkinsonian disorder | 2.33 | 1.72–3.16 | <0.001 |
| Hypertension | 1.14 | 0.97–1.33 | 0.109 |
| Angina pectoris | 0.89 | 0.75–1.05 | 0.163 |
| Atrial fibrillation | 1.22 | 1.00–1.48 | 0.055 |
| Heart failure | 1.21 | 1.03–1.42 | 0.020 |
| Cerebrovascular disease | 1.18 | 1.02–1.37 | 0.028 |
| Pneumonia | 2.40 | 1.95–2.96 | <0.001 |
| Asthma | 1.22 | 1.02–1.45 | 0.031 |
| Gastroesophageal reflux disease | 0.97 | 0.85–1.12 | 0.701 |
| Renal failure | 1.30 | 1.04–1.62 | 0.022 |
| Eating and swallowing dysfunction | 0.70 | 0.44–1.11 | 0.129 |
| Drugs |  |  |  |
| Antiepileptics | 1.13 | 0.92–1.38 | 0.245 |
| Anticholinergics | 1.67 | 0.76–3.66 | 0.203 |
| Hypnotics | 1.00 | 0.86–1.15 | 0.968 |
| Cerebrovascular rehabilitation | 1.06 | 0.66–1.71 | 0.813 |
| Nasal feeding | 3.91 | 1.43–10.66 | 0.010 |

**Online Resource 3.** Detailed results of multivariate Cox regression analysis for all-cause hospitalization.

|  | Hazard ratio | 95% confidence interval | P-value |
| --- | --- | --- | --- |
| Self-reported difficulty in oral function |  |  |  |
| Without any self-reported difficulty in oral function | reference | | |
| Only self-reported difficulty in chewing | 1.08 | 1.05–1.11 | <0.001 |
| Only self-reported difficulty in swallowing | 1.01 | 0.97–1.04 | 0.678 |
| Both self-reported difficulty in chewing and swallowing | 1.12 | 1.09–1.16 | <0.001 |
| Age, years |  |  |  |
| 75–79 | reference | | |
| 80–84 | 1.05 | 1.02–1.07 | <0.001 |
| 85–89 | 1.20 | 1.16–1.23 | <0.001 |
| 90–94 | 1.41 | 1.34–1.48 | <0.001 |
| ≥95 | 1.95 | 1.78–2.14 | <0.001 |
| Male | 1.33 | 1.30–1.36 | <0.001 |
| Body mass index, kg/m^2^ |  |  |  |
| <18.5 | 1.12 | 1.08–1.17 | <0.001 |
| 18.5–24.9 | Reference | | |
| 25.0-29.9 | 1.06 | 1.04–1.09 | <0.001 |
| ≥30.0 | 1.14 | 1.07–1.21 | <0.001 |
| Charlson comorbidity index |  |  |  |
| 0–2 | reference | | |
| 3–4 | 1.16 | 1.13–1.20 | <0.001 |
| ≥5 | 1.30 | 1.23–1.36 | <0.001 |
| Smoking habit |  |  |  |
| Non-smoker | reference | | |
| Current smoker | 1.00 | 0.95–1.04 | 0.878 |
| Past smoker | 1.05 | 1.02–1.08 | 0.002 |
| Polypharmacy |  |  |  |
| No polypharmacy (0–4) | reference | | |
| Polypharmacy (5–9) | 1.13 | 1.10–1.16 | <0.001 |
| Hyper-polypharmacy (≥10) | 1.34 | 1.25–1.45 | <0.001 |
| Comorbid medical conditions |  |  | 0.004 |
| Diabetes | 1.05 | 1.02–1.07 | <0.001 |
| Dementia | 1.14 | 1.10–1.20 | <0.001 |
| Parkinson's disease or Parkinsonian disorder | 1.13 | 1.04–1.23 | <0.001 |
| Hypertension | 1.06 | 1.03–1.09 | <0.001 |
| Angina pectoris | 1.10 | 1.07–1.13 | <0.001 |
| Atrial fibrillation | 1.24 | 1.19–1.28 | <0.001 |
| Heart failure | 1.08 | 1.05–1.11 | 0.602 |
| Cerebrovascular disease | 1.07 | 1.05–1.10 | <0.001 |
| Pneumonia | 1.51 | 1.44–1.58 | 0.001 |
| Asthma | 1.01 | 0.98–1.04 | 0.001 |
| Gastroesophageal reflux disease | 1.15 | 1.12–1.17 | <0.001 |
| Renal failure | 1.18 | 1.13–1.23 | <0.001 |
| Eating and swallowing dysfunction | 0.87 | 0.81–0.94 | 0.004 |
| Drugs |  |  |  |
| Antiepileptics | 1.25 | 1.21–1.30 | 0.001 |
| Anticholinergics | 0.95 | 0.76–1.19 | 0.660 |
| Hypnotics | 1.16 | 1.13–1.18 | <0.001 |
| Cerebrovascular rehabilitation | 1.44 | 1.32–1.57 | <0.001 |
| Nasal feeding | 1.74 | 1.34–2.28 | <0.001 |

**Online Resource 4.** Detailed results of multivariate Cox regression analysis for all-cause mortality.

|  | Hazard ratio | 95% confidence interval | P-value |
| --- | --- | --- | --- |
| Self-reported difficulty in oral function |  |  |  |
| Without any self-reported difficulty in oral function | reference | | |
| Only self-reported difficulty in chewing | 1.28 | 1.14–1.44 | <0.001 |
| Only self-reported difficulty in swallowing | 1.02 | 0.87–1.20 | 0.776 |
| Both self-reported difficulty in chewing and swallowing | 1.75 | 1.53–2.00 | <0.001 |
| Age, years |  |  |  |
| 75–79 | reference | | |
| 80–84 | 1.78 | 1.56–2.20 | <0.001 |
| 85–89 | 3.40 | 2.97–3.89 | <0.001 |
| 90–94 | 5.62 | 4.76–6.64 | <0.001 |
| ≥95 | 12.18 | 9.68–15.32 | <0.001 |
| Male | 2.12 | 1.91–2.36 | <0.001 |
| Body mass index, kg/m^2^ |  |  |  |
| <18.5 | 2.43 | 2.14–2.76 | <0.001 |
| 18.5–24.9 | reference | | |
| 25.0-29.9 | 0.78 | 0.68–0.88 | <0.001 |
| ≥30.0 | 0.86 | 0.62–1.21 | 0.393 |
| Charlson comorbidity index |  |  |  |
| 0–2 | reference | | |
| 3–4 | 1.29 | 1.13–1.47 | <0.001 |
| ≥5 | 1.36 | 1.11–1.67 | 0.003 |
| Smoking habit |  |  |  |
| Non-smoker | reference | | |
| Current smoker | 1.48 | 1.24–1.76 | <0.001 |
| Past smoker | 1.05 | 0.93–1.18 | 0.467 |
| Polypharmacy |  |  |  |
| No polypharmacy (0–4) | reference | | |
| Polypharmacy (5–9) | 1.18 | 1.05–1.32 | 0.005 |
| Hyper-polypharmacy (≥10) | 1.78 | 1.33–2.38 | <0.001 |
| Comorbid medical conditions |  |  |  |
| Diabetes | 1.22 | 1.10–1.35 | <0.001 |
| Dementia | 1.88 | 1.63–2.17 | <0.001 |
| Parkinson's disease or Parkinsonian disorder | 1.58 | 1.19–2.10 | 0.002 |
| Hypertension | 1.06 | 0.95–1.19 | 0.309 |
| Angina pectoris | 0.95 | 0.84–1.07 | 0.384 |
| Atrial fibrillation | 1.22 | 1.04–1.42 | 0.012 |
| Heart failure | 1.13 | 1.00–1.27 | 0.056 |
| Cerebrovascular disease | 0.92 | 0.82–1.04 | 0.173 |
| Pneumonia | 2.34 | 1.99–2.74 | <0.001 |
| Asthma | 0.91 | 0.79–1.05 | 0.197 |
| Gastroesophageal reflux disease | 1.05 | 0.95–1.16 | 0.346 |
| Renal failure | 1.33 | 1.13–1.57 | 0.001 |
| Eating and swallowing dysfunction | 1.13 | 0.85–1.52 | 0.398 |
| Drugs |  |  |  |
| Antiepileptics | 1.09 | 0.93–1.28 | 0.277 |
| Anticholinergics | 0.74 | 0.27–2.03 | 0.561 |
| Hypnotics | 0.88 | 0.79–0.98 | 0.025 |
| Cerebrovascular rehabilitation | 0.96 | 0.64–1.43 | 0.825 |
| Nasal feeding | 2.25 | 0.83–6.10 | 0.109 |
